# Recent Advances in Strategies Against Measles Spread: Clinical and Epidemiological Aspects, Impact, Vaccine Innovations, and Sustainable Recommendations

**DOI:** 10.1155/tswj/9008185

**Published:** 2026-04-26

**Authors:** Abdelilah Merabti, Youssef Miyah, Mohammed Benjelloun, Chadia Zahouani, Wafaa Al Hassani, Rajae Lamsyah, Samia Essadki, Laila Ihrai, Saadia El Filali, Anis Sfendla, Mustapha Yaagoubi, Chakib Nejjari

**Affiliations:** ^1^ Higher Institute of Nursing Professions and Health Techniques, Ministry of Health and Social Protection, Fez, Morocco; ^2^ Laboratory of Materials, Processes, Catalysis, Agri-Food, and Environment, Higher School of Technology, Sidi Mohamed Ben Abdellah University, Fez, Morocco, usmba.ac.ma; ^3^ Laboratory of Natural Resources and Economics of Sustainable Development, Polydisciplinary Faculty of Larache, Abdelmalek Essaadi University, Tetouan, Morocco, uae.ma; ^4^ Euromed Research Center, Euromed University of Fez, Fez, Morocco; ^5^ Higher Institute of Nursing Professions and Health Techniques, Ministry of Health and Social Protection, Oujda, Morocco; ^6^ Maternal Child and Mental Health Research Laboratory, Faculty of Medicine and Pharmacy of Oujda, Mohammed First University, Oujda, Morocco; ^7^ Laboratory of Applied Human Sciences, Higher Normal School (ENS), Sidi Mohamed Ben Abdellah University, Fez, Morocco, usmba.ac.ma; ^8^ Faculty of Medicine, Pharmacy, and Dentistry, Sidi Mohamed Ben Abdellah University, Fez, Morocco, usmba.ac.ma

**Keywords:** diet, epidemiology, health, immunity, measles, vaccine

## Abstract

**Objectives:**

Measles is a highly contagious disease, transmitted by respiratory droplets, whose spread is favored by disparities in vaccination coverage. This manuscript analyzes recent measles control strategies, analyzing their evolution, environmental repercussions, immune mechanisms, innovations in immunization, and sustainable solutions.

**Study Design:**

Measles harms public health, and the socioeconomic landscape and advances in smart technologies offer innovative solutions for combating the disease.

**Methods:**

Obstacles to the fight against measles include low vaccination coverage, community resistance, and logistical problems related to vaccine distribution.

**Results:**

The immune response to measles involves B and T cells, which are crucial in eliminating the virus and forming an immune memory. Improvements in measles vaccines include the development of genetically modified vaccines and advanced delivery technologies.

**Conclusions:**

To eliminate measles, the manuscript recommends exploring data‐driven and digital surveillance tools as complementary support to traditional epidemiological systems.

## 1. Introduction

Despite the sharp reduction in measles cases, thanks to the integration of the combined measles, mumps, and rubella vaccine into immunization programs, this disease continues to represent a major threat to child health worldwide, still leading to high morbidity and mortality [1]. With an estimated basic reproduction rate of between 12 and 18, measles is one of the most contagious diseases, as a single person can infect up to 18 others in the absence of herd immunity, making high vaccination coverage essential to limit its spread, although its elimination remains uneven across regions despite an effective vaccine [2]. Without this generalized protection, the slightest laxity in vaccination can lead to epidemic outbreaks, endangering vulnerable populations, particularly unvaccinated infants and immunocompromised individuals [3]. This spread has been attributed to disparities in vaccination rates, the emergence of antivaccine movements, and the inability of certain healthcare systems to reach at‐risk groups [4]. Globalization and increased human mobility have also contributed to the transmission of the virus between different countries and communities, complicating efforts to contain the disease. Vaccines are the most effective means of reducing the spread of measles, but vaccination coverage remains insufficient worldwide, particularly in poor countries and those in political crisis [5, 6]. Over the last few decades, misinformation about the side effects of vaccines has become widespread, leading some communities to refuse vaccination, thus creating environments conducive to the outbreak of disease [7]. In addition, many low‐income countries suffer from a lack of medical resources, which makes it difficult to effectively implement vaccination programs or rapidly monitor suspected cases [8]. Moreover, with increased human mobility across borders, the virus is easily transmitted from one region to another, facilitating the emergence of new cases in previously spared areas [9]. In addition, social factors such as poor diet and immunity weakened by chronic disease increase the risk of developing complications [10].

Measles, although an acute, highly contagious disease caused by a virus of the Paramyxoviridae family, is spread by respiratory droplets suspended in the air or on surfaces and initially manifests itself as fever, cough, runny nose, and conjunctivitis, followed by a rash [11]. Measles has evolved significantly since its recognition as a distinct disease, but the fight against the disease remains a global challenge, especially against a backdrop of antivaccination movements and logistical difficulties in certain regions [12]. Despite widespread vaccination, measles persists due to insufficient vaccination rates, low herd immunity, and regional disparities [13]. Obstacles to measles treatment are multiple and complex, including limited access to the vaccine in low‐income countries, the rise of antivaccination movements that promote misinformation and decrease vaccination coverage, and disruptions to vaccination campaigns due to global crises such as the COVID‐19 pandemic [14]. In addition, logistical challenges in remote areas, such as the lack of infrastructure and resources to distribute vaccines, further complicate the situation [15].

The COVID‐19 pandemic significantly disrupted routine immunization services across Africa, leading to substantial declines in measles vaccine coverage and increasing the risk of resurgence outbreaks in multiple countries [16].

Strengthening measles control requires a combination of evidence‐based vaccination strategies, resilient health infrastructures, and improved surveillance systems. In this context, digital epidemiological tools have been explored as complementary approaches to support outbreak prediction and resource allocation [17]. However, their effectiveness depends on data quality, governance frameworks, and feasibility within local health system capacities. It is also necessary to develop more affordable and better‐tolerated vaccines while strengthening international cooperation to finance and improve healthcare services [18].

To ensure the sustainability of these efforts, it is necessary to adopt a comprehensive approach that combines the promotion of sustainable immunization programs, the improvement of health infrastructures through innovation and technology, and the raising of community awareness through effective and innovative means of communication [19]. Analysis of the epidemiological, social, and environmental factors influencing the spread of measles enables us to design intelligent, progressive health policies capable of adapting to change and ensuring long‐term prevention [20].

This manuscript aims to analyze recent measles control strategies, analyzing their geographical and historical evolution, societal and environmental repercussions, immune mechanisms and means of protection, and innovations in immunization and sustainable solutions. By shedding light on the various aspects of this subject, it is possible to propose sustainable solutions that help minimize the spread of the disease, thereby protecting the health of individuals and communities on a large scale. The added value of this review lies in integrating emerging digital surveillance approaches within established measles control frameworks, while critically examining their feasibility and limitations. It also highlights the importance of nutrition in boosting the immunity of affected children, presenting key foods that promote recovery. The review recommends the introduction of a third dose of vaccine to boost protection, particularly in regions with low vaccination coverage, and stresses the importance of raising parents’ awareness of vaccination to prevent epidemics. The article analyzes the evolution of measles control strategies, their impact on the education and psychology of children and their parents, as well as the immune mechanisms underlying the vaccine and its efficacy. It also explores recent innovations in the design of new vaccines and proposes sustainable recommendations for improving measles control in developing countries. This study is of significant importance, given the major health challenges that most countries around the world have faced in recent years due to the measles epidemic.

For clarity, in this manuscript, “elimination” refers to the interruption of endemic transmission in a defined geographic area requiring ongoing surveillance, whereas “eradication” denotes the permanent global cessation of transmission. The “herd immunity threshold” represents the theoretical minimum level of population immunity required to prevent sustained transmission. “Modified measles” refers to attenuated or atypical disease presentation in partially immune individuals.

## 2. Drivers of Measles Resurgence

### 2.1. Increase in Measles Cases in Some Regions of the World

**FIGURE 1:**
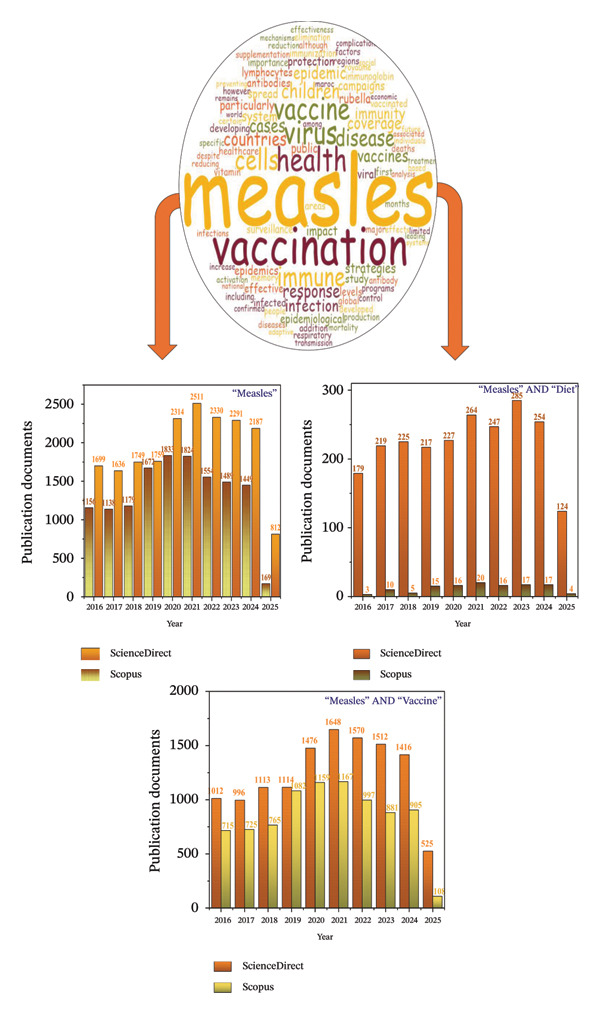
Word cloud and bibliometry of measles in relation to diet and vaccine (original figure created by authors).

Although significant progress has been made in making measles vaccines available, and many health programs have succeeded in reducing the spread of the disease, there has been a marked increase in measles cases in some regions of the world. This increase in measles cases is a public health problem affecting the health of many communities. It requires urgent and effective intervention to tackle the causes of the resurgence of this infectious disease [21]. This interest could be expressed in the increased annual publications in search engines and databases (Figure 1).

While the development of the measles vaccine stands as one of the most significant medical advancements of the 20th century, the precise causes behind the recent resurgence of the disease remain poorly understood [22]. Although vaccines are available in many countries, multiple factors play an important role in the continued spread of the disease, including social transformations in some societies, inconsistent or ineffective health policies in implementing vaccination programs, and cultural and social barriers that prevent individuals from making informed decisions about measles vaccination.

Socio‐economic transitions, such as armed conflict or mass migration, can alter the lifestyles and health behaviors of many people, leading to a deterioration in vaccination coverage and a resurgence of measles. Inconsistent or unsystematic health policies, whether at the state or local level, can reduce the effectiveness of vaccination campaigns.

Analysis of the cultural and social barriers that prevent individuals from accepting vaccinations is a key element of this study. In some societies, there may be cultural resistance to vaccination due to myths or misconceptions about vaccines, leading to a lack of trust in government health programs. This lack of trust can be exacerbated by global health crises such as the COVID‐19 pandemic, which demonstrated the vulnerability of healthcare systems in some countries and had a significant impact on measles vaccination strategies [23, 24].

In addition, the study seeks to provide a comprehensive analysis based on statistical data from various geographical regions, enabling regional factors and variations in disease prevalence rates to be investigated. Through this multidimensional analysis, the study hopes to provide a clear picture of the influencing factors contributing to the increase in measles cases. The study aims to contribute to the development of practical solutions for targeting groups most at risk from measles, such as children in rural areas, or populations suffering from severe poverty or with limited access to basic healthcare services.

This study should help to identify the main constraints hindering the effectiveness of current prevention strategies, such as the difficulty of reaching remote areas, where there is no nearby health center, or where local health systems encounter difficulties in distributing vaccines. In addition, the lack of awareness among many populations of the importance of measles vaccination will be highlighted. In some cases, there is still a lack of education and awareness of the benefits of vaccines, making some people reluctant to make the decision to be vaccinated [25].

About the COVID‐19 pandemic, the impact of this global health crisis on measles vaccination campaigns will be highlighted. The pandemic has disrupted many global health activities, including routine vaccination campaigns [26]. The vaccine supply chain was disrupted, leading to delays in vaccine delivery in many countries. Preoccupation with COVID‐19 also led some governments and health organizations to lose interest in combating other diseases such as measles, contributing to the spread of the pandemic in certain regions [27].

This review synthesizes programmatic strategies for measles control, emerging vaccine technologies to identify practical approaches to strengthen public health responses. By integrating current evidence, it aims to inform policy and optimize vaccination strategies to reduce transmission and advance measles elimination efforts.

### 2.2. Global Epidemiological Situation

Measles, a highly contagious viral disease, represents a major public health challenge. It often starts with an infection of the respiratory tract before compromising other vital organs, leading to severe complications and, in some cases, death. Although the disease can occur at any age, young children are the most frequently affected [28].

Between January and August 2020, more than 1.3 million children in Africa missed their first measles vaccine dose compared to the same period in 2019, raising concerns about large‐scale outbreaks [16]. For instance, Morocco is considered a country with a national immunization program, considered excellent by the WHO. However, the COVID‐19 pandemic has led to a worrying decline in vaccination rates worldwide, exposing millions of children to the risk of measles, triggering fulminant epidemics, and hampering the achievement of measles elimination targets [29–31]. In Morocco, since the advent of the COVID‐19 pandemic, the situation has been marked by a drop in vaccination coverage and already two measles outbreaks in the Béni Mellal‐Khénifra region in the first half of 2023 [32]. In Morocco, a measles epidemic broke out in September 2023 in three localities north of Agadir‐Idda Outanane, before spreading rapidly to the various prefectures and provinces of the Souss‐Massa region, then to those of Marrakech‐Safi, and then to the rest of the country [33]. The current measles epidemic began in the Souss‐Massa region and then gradually spread to neighboring provinces in the Marrakech‐Safi region, before affecting all regions of the Kingdom [34].

The 2022 measles outbreak in Zimbabwe resulted in over 2000 confirmed cases and more than 150 deaths, largely affecting under‐immunized communities [35]. The outbreak highlighted the consequences of delayed immunization campaigns and vaccine refusal in vulnerable populations.

### 2.3. Historical and Geographical Approaches to Measles Control

Throughout history, measles epidemics have had devastating health consequences, especially in regions with low vaccination coverage. Measles is highly contagious and has had a lasting impact on global public health [36]. Different strategies have been put in place to combat the disease, ranging from quarantine to large‐scale vaccination campaigns, transforming the management of measles, particularly in developed countries. Measles epidemics highlight the serious consequences of the disease, particularly for vulnerable populations [37]. Management of measles varies according to a country’s level of development. Developed countries have succeeded in considerably reducing the number of cases, thanks to widespread vaccination programs. However, measles remains a persistent threat in developing and underdeveloped countries, due to insufficient vaccination coverage and limited access to healthcare [38]. Public health measures such as quarantine, patient isolation, and mass vaccination campaigns were implemented to reduce the incidence and mortality of the disease. The history of measles is characterized by major epidemics that had far‐reaching consequences before the introduction of vaccination [39]. Countries have adopted different strategies to manage the disease, as demonstrated by Nepal’s response in 2020 [40]. Measles remains a persistent threat, causing waves of morbidity and mortality, particularly in regions with low vaccination coverage. The management of measles is influenced by geographical and economic context, with developed countries having succeeded in reducing the number of cases, thanks to sustained vaccination programs, while developing and underdeveloped countries face challenges in terms of access to vaccines and health structures [41]. Consequently, the history of major measles epidemics demonstrates the devastating impact of this highly contagious disease, particularly before the introduction of vaccines (Table 1). Effective management strategies, such as vaccination campaigns and public health interventions, have helped to reduce the number of cases and improve outcomes. However, ongoing efforts are needed to ensure universal access to vaccines and health services to control and ultimately eliminate measles worldwide. The study conducted by Adam et al. in Sudan in 2015–2017 shows a high seroprevalence of measles antibodies in children and adults, suggesting an effective control program but highlights active circulation of mumps and rubella viruses. These results illustrate the impact of public health strategies adopted over time, notably quarantine and vaccination, which have helped to contain the disease in several regions of the world [48]. The study of respiratory infections during the Hajj in 2014 illustrates the challenges posed by the transmission of infectious diseases in contexts of high human concentration, despite vaccine recommendations; although the efficacy of certain vaccines has been demonstrated, their adoption remains uneven across demographic groups [49]. The study carried out by Al‐Ghamdi et al. in Saudi Arabia on the Tabuk epidemic highlights the disparities in vaccination by age and the vaccine failure rate (35%), underlining the need for optimal vaccine coverage. It also shows that vaccinated individuals had a significantly reduced risk of contracting measles compared with nonvaccinated individuals. This analysis is in line with the surveillance strategies implemented in many countries to monitor the evolution of cases and adjust public health responses. Historically, before the advent of vaccines, traditional methods were used, albeit with limited efficacy, and epidemic management relied mainly on isolating patients. This Saudi study in Tabuk illustrates the importance of epidemiological surveillance systems in preventing outbreaks and reinforcing vaccination policies, particularly in developing countries where measles elimination remains a major challenge [50]. The study by Amendola et al. on molecular surveillance of measles virus genotype D8 in Northern Italy between 2013 and 2014 illustrates the complexity of transmission, with several independent chains and a persistent endemic strain. This situation reflects the challenges encountered in various geographical contexts: While developed countries, such as Italy, have advanced tools for tracing virus circulation and assessing the effectiveness of vaccination strategies, developing and underdeveloped countries face structural obstacles hindering vaccination coverage [51]. Atkinson et al. claim that measles, an acute viral disease, has a marked history with major epidemics documented since the 7th century, with a detailed description by Rhazes in the 10th century calling it more fearsome than smallpox. In 1846, Panum identified its incubation period and the lifelong immunity it confers. The isolation of the virus in 1954 by Enders and Peebles paved the way for the development of the first attenuated vaccine in 1963 (Edmonston B strain), marking a decisive turning point in the fight against the disease. Before the vaccine era, measles affected between 3 and 4 million people a year in the United States, with epidemic cycles occurring every 2–3 years, high incidence in children aged 5 to 9, and high mortality in those under 5. The introduction of the vaccine led to a 98% reduction in cases and the disappearance of epidemic cycles. Elimination programs, such as the one launched in 1978 in the United States, led to an all‐time low of 1497 cases in 1983, although fluctuations were subsequently observed [42]. Auzenbergs et al. claim that an analysis of 14 countries (India, Nigeria, Indonesia, Ethiopia, China, Philippines, Uganda, Democratic Republic of Congo, Pakistan, Angola, Madagascar, Ukraine, Malawi, and Somalia) with high measles incidence, accounting for 53% of global births and 78% of the global measles burden, revealed that the introduction of the first vaccine dose prevented 824 million cases and 9.6 million deaths, while the addition of the second vaccine dose and supplementary immunization campaigns boosted protection, although the latter was less effective due to the diversity of ages targeted [52]. Bahri et al. consider that the introduction of the measles vaccine in Tunisia in 1979 marked a decisive turning point in the management of this disease, illustrating the evolution of control approaches across history and geographical contexts. In the past, major measles epidemics led to high infant mortality, severe complications, and sanitary overload, particularly in countries with limited resources, where traditional methods such as herbal remedies and isolation of patients were the only available responses. Analysis of Tunisian data between 1979 and 2000 highlights the impact of vaccination campaigns in reducing the incidence and rarity of outbreaks after 1992, with vaccination coverage exceeding 90% from that date. This model is in line with the strategies adopted in many developed countries, where mass vaccination and epidemiological surveillance have led to the virtual elimination of the disease, while in developing countries, limited access to vaccines and health infrastructures further complicates case management [44]. Berche reports that from the first clinical description of measles by Rhazes in the 10th century to its virological identification by Peebles and Enders in 1954, measles has a marked medical history of its high contagiousness and mortality, particularly, in developing countries where it still causes over 100,000 deaths annually. The global spread of the disease since the Renaissance has given rise to major epidemiological studies, such as Peter Panum’s study of the 1846 epidemic in the Faroe Islands. The discovery of the viral nature of measles in 1911 paved the way for the development of a live attenuated vaccine in 1958, licensed in the United States in 1963, which revolutionized the fight against the disease, thanks to a large‐scale vaccination campaign led by the WHO. However, disparities persist between developed countries, where vaccination has virtually eradicated measles, and low‐resource countries, particularly, in Africa, South America, and Asia, where the disease remains a major public health challenge [43]. Despite the COVID‐19 pandemic, Nepal’s National Immunization Program has implemented a reactive vaccination campaign in addition to a preventive campaign against measles and rubella [47]. Faced with an outbreak of 220 cases and two deaths, an approach based on the analysis of epidemiological and surveillance data made it possible to target interventions, resulting in the vaccination of over 32,000 people with a coverage rate of 97%, leading to a 98% reduction in the incidence of measles [47]. This exemplary management, supported by the World Health Organization and the United Nations Children’s Fund, demonstrates the importance of data‐driven strategic decisions and interinstitutional collaboration in the fight against measles, reflecting a historical evolution of public health responses, from traditional remedies to rigorously planned vaccine campaigns. The study by Boulton et al. in Tianjin, China, shows that despite high vaccine coverage, the herd immunity needed to interrupt transmission is not being achieved, particularly in adults aged 20–39, highlighting the need to strengthen immunization strategies for the whole population [53]. Dayan and McLean assert that, despite the availability of an effective vaccine for over 50 years, measles remains the leading cause of vaccine‐preventable infant mortality [45]. Dayan and McLean report that in 2012, there were an estimated 122,000 measles deaths worldwide. Over 95% of measles deaths occur in developing countries [45]. The World Health Organization’s strategy for reducing measles mortality is to achieve and maintain high levels of population immunity by ensuring high vaccination coverage with two doses of measles vaccine. Dayan and McLean state that between 2000 and 2012, improving routine immunization coverage and implementing supplementary measles immunization activities led to a 78% reduction in the estimated number of measles deaths worldwide [45]. Ding et al. report that the study of 284 confirmed cases of measles in Tianjin, China, reveals that postvaccine immunity can weaken over time, leading to secondary vaccine failures, although these cases generally remain less severe than those with low antibody avidity [54]. Do et al. suggest that the 2013–2014 measles epidemic in Vietnam illustrates the persistent challenges in combating this disease, particularly in populations with low vaccination coverage? With 9577 confirmed cases and a predominance of infections in children under five, this epidemic initially broke out in a minority living in isolated mountainous areas, before spreading to densely populated urban centers. The cocirculation of H1 and D8 strains of the virus, some of which were genetically close to those detected in China and Laos, underlines the importance of cross‐border exchanges in the spread of the virus. The evolution of public health responses to measles has seen the gradual adoption of vaccination as the main preventive tool, replacing older strategies such as quarantine or traditional treatments, which are often ineffective. This episode highlights the need to adapt vaccination campaigns to local realities and to strengthen epidemiological surveillance to better contain and prevent future measles outbreaks [46]. Dunn et al. report that in the first half of 2019, more than 182 countries reported over 300,000 cases of measles, more than double compared to the same period in 2018 [55]. Timely recognition and laboratory confirmation of infected individuals, together with appropriate infection prevention measures, are crucial to prevent further transmission [55]. Durrheim et al. suggest that public health responses have evolved from quarantine and traditional symptomatic treatments to large‐scale vaccination campaigns, supported by initiatives such as the Global Vaccine Action Plan, which aims to eliminate measles in at least five of the six WHO regions. Measles epidemiology provides valuable data on predictable transmission patterns, epidemic seasonality, and the effectiveness of control strategies, as demonstrated by analyses carried out in the Americas and Western Pacific Regions, where surveillance and verification mechanisms have been set up to monitor progress toward the elimination of the disease [56]. The study by Fu et al. of the measles epidemic in a Chinese medicine hospital in Beijing, 2018 illustrates the persistence of epidemic outbreaks despite the elimination efforts undertaken in China since 2006 [57]. This epidemic, which mainly affected unvaccinated healthcare workers or those with unknown vaccination status, highlights the crucial role of vaccination coverage in preventing nosocomial transmission, particularly, in healthcare facilities. Analysis of the cases revealed that a lack of vaccination documentation was a major risk factor, underlining the importance of immunization monitoring for healthcare workers. The health response, based on the isolation of cases, increased surveillance, and the administration of more than 3200 doses of vaccine in the hospital and the community made it possible to contain the spread of the virus, demonstrating the effectiveness of targeted vaccination campaigns [57]. This case study reflects the persistent challenges associated with measles, even in countries with ambitious elimination plans, and serves as a reminder of the importance of systematic immunization of healthcare workers to prevent nosocomial transmission [57]. Fujisaki et al.’s study of the 2007 epidemic at Teikyo University shows that antibody screening and selective vaccination of students with low immunity enabled the epidemic to be contained, with no new cases following the implementation of the vaccination program, illustrating the effectiveness of modern strategies [58]. González‐Praetorius et al. describe a measles epidemic that occurred in 2019 in the province of Guadalajara, Spain, highlighting the challenges of microbiological diagnosis in the context of disease elimination [59]. Historically, public health responses have evolved from quarantine and symptomatic treatment to the mass adoption of vaccination, now the key elimination strategy. The Spanish study illustrates the importance of accurate diagnosis, combining molecular testing and detection of specific immunoglobulin M, to distinguish natural infections from postvaccination cases, underlining the essential role of genotyping in measles control efforts [59]. Hanson et al. highlight the safety of the measles, mumps, and rubella vaccine in adolescents and adults, observing that serious side effects remain rare and that nonserious effects, such as fever and injection site reactions, are more frequent but generally mild [60]. In the past, traditional methods were used to treat measles, but their effectiveness was limited compared with modern interventions such as vaccines, which have radically changed the global approach to disease prevention [60]. The study by Ho et al. on measles in Singapore highlights the historical and geographical evolution of the control of this disease, through the analysis of epidemiological trends from 1981 to 2012 [61]. Initially, measles had a high incidence rate, reaching 88.5 cases per 100,000 inhabitants in 1984, but thanks to the successful implementation of the National Childhood Vaccination Program, incidence fell considerably to 6.9 cases per 100,000 in 1991 [61]. However, resurgences occurred in 1992, 1993, and 1997, before the introduction of a catch‐up vaccination program with trivalent measles, mumps, and rubella vaccine in 1997, followed by the introduction of a two‐dose vaccination schedule in 1998 [61]. The results were remarkable, with incidence falling to 2.9 per 100,000 in 1998, while vaccination coverage reached 95% for the first dose and 92%–94% for the second. However, sporadic cases and clusters persisted among unvaccinated populations, especially among children under the age of 4 [61]. Geographically, measles virus strains have evolved from the D9 genotype to B3 and G3 from neighboring countries. [61]. Historically, public health responses have evolved from quarantine to increasingly targeted vaccination campaigns. In addition to vaccination, traditional measles treatment methods used in the past focused mainly on herbal remedies and homecare practices, which have gradually given way to modern medical interventions. As a result, measles management in Singapore has evolved significantly, with epidemiologically based preventive strategies and increasing vaccination coverage contributing to the gradual elimination of the disease [61]. Hangerford et al. relate the 2012 measles epidemic in Merseyside, UK, as a striking example, where 359 cases were confirmed. This case study revealed that risk factors associated with measles transmission included incomplete vaccinations, age below that recommended for vaccination, and hospitalizations [62]. The measles epidemics observed in Georgia during the 2004–2005 and 2013–2015 crises showed not only the immediate impact of the disease but also the long‐term risks, such as subacute sclerosing panencephalitis, a fatal complication that is often overlooked [63]. During these epidemics, 8377 and 11,495 cases of measles were recorded, but the burden of subacute sclerosing panencephalitis has not been sufficiently studied [63]. By analyzing cases of subacute sclerosing panencephalitis between 2008 and 2017, we estimated that the risk of subacute sclerosing panencephalitis for the 2004–2005 epidemic was one case per 1,396, with adjustments to account for under‐registration of measles cases [63]. This risk demonstrates an increased need to boost vaccination coverage in Georgia, particularly in children, and to fill immunity gaps in adults. Kurata et al. say that in Japan, a major turning point came with the launch of vaccination in 1978, epidemiological monitoring in 1981, and the elimination of the disease in 2015, following improvements in surveillance methods and vaccine programs [64]. A longitudinal study conducted in Osaka, Japan, between 1982 and 2021 revealed that vaccination achieved coverage of over 95% for the first dose as early as 1998 and 90% for the second dose in 2009, with a seroprevalence of over 95% in 2011 [64]. However, the reduced incidence of the disease has led to a decline in antibody titers, making certain populations vulnerable to vaccine failure and the appearance of modified forms of measles. This phenomenon, particularly, observed after the elimination of the disease in 2015, revealed genotypes derived from imported cases, underlining the need for rigorous surveillance of herd immunity [64]. Marchi et al. examined the prevalence of measles antibodies in Italy between 1993 and 2018, highlighting the impact of mass vaccination campaigns and public health strategies implemented over time [65]. In Italy, the inclusion of the vaccine in vaccination schedules and national campaigns has improved vaccination coverage, but epidemics continue to affect mainly adolescents and adults. The study revealed that, despite the increase in vaccination coverage, a significant proportion of the population remains vulnerable, particularly in older age groups, suggesting the need for policies that also target the adult population to fill immunity gaps and achieve the goal of eliminating the disease [65]. Muscat et al. highlight the impact of major measles epidemics in Europe between 2006 and 2007, with a total of 12,132 cases recorded over 2 years, 85% of which came from five countries: Romania, Germany, the United Kingdom, Switzerland, and Italy. The cases were mainly unvaccinated or poorly vaccinated children, but almost a fifth of patients were aged 20 or over [66]. Rees reports that measles, often confused with other similar diseases, was identified as early as the fifth century BC, but it was not until the 17th and 18th centuries that it was distinguished from these diseases, and reliable diagnostic methods only became available at the end of the 19th century and the measles virus was isolated and characterized in 1950 [67]. Measles epidemics have had a significant impact on health over time, and the response to these crises has varied from country to country and from period to period. Public health strategies such as quarantine and vaccination campaigns have been used [67]. Rees reports that developed countries implemented large‐scale vaccination programs during the 20th century, while developing and underdeveloped countries had limited access to these vaccines, resulting in higher mortality rates [67]. Smith et al. examine the measles epidemic that struck Kent State University in 1989, one of the most severe in the United States before the disease was eliminated in 2000 [68]. After this epidemic, which revealed gaps in vaccination coverage, the authorities introduced a two‐dose vaccination to eradicate measles. At Kent State, the first cases were reported in October 1988, and although the epidemic slowed during the winter vacations, new cases emerged in February 1989, leading to the isolation of infected students, mandatory vaccination of student groups, and the restriction of public events [68]. By March 1989, the epidemic had grown in scale but was brought under control by a combination of vaccination, quarantine, and suppression of public gatherings [68]. By April 1989, 380 cases had been recorded, and 7000 students had been vaccinated [68]. Smith states that measles management has evolved, particularly in developed countries where more targeted strategies have been adopted, contrasting with the challenges faced in developing and underdeveloped countries where health infrastructures remain insufficient to guarantee widespread access to vaccination. In addition, the management of measles throughout history has often involved more traditional methods, such as treatments based on folk remedies, before modern approaches based on vaccination and quarantine took over [68]. Wolfson et al. revealed that, between 1999 and 2005, measles mortality fell by 60%, from 873,000 deaths with uncertainty limits between 634,000 and 1,140,000 in 1999 to 345,000 deaths with uncertainty limits between 247,000 and 458,000 in 2005 [69]. The greatest reduction in estimated measles mortality over this period was seen in the Western Pacific Region (81%), followed by Africa (75%) and the Eastern Mediterranean region (62%) [69]. Africa recorded the greatest total reduction, contributing 72% of the global reduction in measles mortality [69]. Between 1999 and 2005, nearly 7.5 million measles deaths were prevented by vaccination, with additional immunization activities and improved routine immunization contributing to the prevention of 2.3 million of these deaths [69].

**TABLE 1:** Historical evolution of measles.

Period	Main results	References
Historical	Measles has evolved from an epidemic to an effective vaccine since the virus was discovered in 1954; there has been a 98% reduction in cases after the introduction of the vaccine.	[42]
Historical	Impact of measles through the ages: The role of vaccines in elimination in developed countries and challenges persist in developing countries.	[43]
1979–2000	Impact of measles vaccine introduction in Tunisia; > 90% vaccination coverage after 1992; successful reduction in epidemics.	[44]
2012	122,000 measles deaths; 95% of deaths in developing countries. Improved immunization coverage reduced deaths by 78%.	[45]
2013–2014	Measles outbreak in Vietnam among children under 5 years of age; challenges to disease control in low‐coverage areas.	[46]
2020	Reactive measles and rubella vaccination campaign in Nepal after the COVID‐19 pandemic; 98% reduction in measles cases.	[47]

Throughout history, various sanitary measures have been put in place to manage measles epidemics, ranging from quarantine to traditional treatments and, today, vaccination policies. Traditional remedies such as medicinal plants and isolation were once favored but proved ineffective in the face of the virus’s rapid transmission [70, 71]. Vaccination is now considered an essential strategy for eliminating the disease, although its effectiveness may vary according to social and economic contexts. The persistence of measles outbreaks in certain regions underlines the need for rigorous surveillance and continued vaccination efforts to achieve elimination. Consequently, the evolution of health responses has underlined the long‐term importance of vaccination policies in the fight against measles. The World Health Organization states that measles vaccination prevented more than 60 million deaths between 2000 and 2023 and that although a safe and cost‐effective vaccine is available, there were an estimated 107,500 measles deaths worldwide in 2023, mainly among unvaccinated or undervaccinated children under five, and that the proportion of children who received a first dose of measles vaccine was 83% in 2023, well below the 86% recorded in 2019 [72].

### 2.4. Obstacles to Measles Elimination

The rapid spread of measles makes it imperative to adopt rapid, effective response strategies for its prevention. It has been shown that most children who contract measles develop symptoms only a few days after infection, which means that they can transmit the virus to others before they can receive treatment [28]. Consequently, eliminating measles requires strategies aimed at preventing the transmission of the virus as early as possible, which in turn requires a robust healthcare system capable of treating cases rapidly. One of the main obstacles to eliminating measles is the wide gap in vaccination coverage between different countries. Many developing countries face considerable difficulties in ensuring that all children receive the necessary measles vaccines [73]. In some countries, it can be difficult to supply vaccines in sufficient quantities, particularly, in remote areas or during times of crisis. Inadequate infrastructure can make it difficult to deliver vaccines to communities living in remote areas. In some poor regions, the cost of vaccines or associated healthcare may be too high for families. In some rural or remote areas, it is difficult for vaccination teams to reach households due to a lack of transport or geographical obstacles [74, 75]. Measles resurgence is not confined to low‐income settings. In 2022, an outbreak in Ohio primarily affected unvaccinated children, underscoring the role of vaccine hesitancy even in high‐income countries [76]. Vaccine hesitancy in Africa is influenced by misinformation, mistrust in health systems, political factors, and sociocultural beliefs, requiring context‐specific communication strategies [77]. In many developing regions, the population is extremely poor, making access to healthcare impossible for many families. In these conditions, vaccination against viral diseases such as measles is not accessible to many children. In some societies, there may be resistance to vaccination due to cultural or religious beliefs. Some may consider that the vaccine conflicts with their religious beliefs or practices, resulting in low vaccination coverage. In some cases, political conflict or war can hamper vaccination campaigns. For example, in regions affected by armed conflict, such as certain countries in the Middle East, conflict can disrupt vaccination programs and increase the risk of measles epidemics [78, 79]. Inadequate health infrastructures are another obstacle to eliminating measles. We know that vaccines require certain storage conditions, such as low temperatures, to retain their effectiveness. These conditions can be difficult to achieve in areas where infrastructure is poor or where there are power cuts [80]. In many developing countries, there is a shortage of medical staff trained in the proper conduct of vaccination campaigns, making it difficult to implement them effectively. In some regions, there are no accurate health information systems to track who has been vaccinated and ensure universal coverage. In some regions, there are no precise health information systems to know who has been vaccinated and to ensure universal coverage. Misinformation is a further complicating factor in the fight against measles. For years, some antivaccination groups have been spreading misinformation about the potential risks of vaccines, including claims that they can cause autism [80]. This inaccurate information creates fear and skepticism among parents, increasing their reluctance to have their children vaccinated. Responding to this problem requires major community awareness‐raising efforts, as well as the provision of accurate and reliable information through official and social media. In addition, governments and global health organizations need to promote confidence in vaccines by highlighting the benefits of vaccination and the risks of not vaccinating. In many countries, measles vaccination programs come up against other health priorities. For example, during the COVID‐19 pandemic, the top priority of many governments and health institutions was to tackle the pandemic, which reduced the resources devoted to combating other diseases such as measles [81]. Too much focus on other diseases such as malaria and tuberculosis can reduce the attention needed for immunization programs, slowing down measles elimination efforts, which is why governments and international health organizations must ensure that measles control does not remain on the sidelines of major health crises. Herd immunity is one of the most important foundations of measles control strategies. Preventing the disease requires a high proportion of the population to be vaccinated, to ensure that the virus is not transmitted from one individual to another. If not enough people are vaccinated, the virus can easily reappear and spread. Undervaccination increases the risk of epidemics, particularly in communities where vaccination rates are low [82]. Although the measles virus is generally genetically stable, it is always possible that mutations may affect the virus’ ability to interact with vaccines. Although current data indicate that such mutations are rare, ongoing monitoring of the virus is essential to ensure the continued efficacy of vaccines. The Moroccan and Arab region faces multiple challenges in vaccine distribution, particularly in rural and remote areas [83]. There are also cultural issues affecting the acceptance of vaccinations. Developing and underdeveloped countries are more exposed to economic and health problems that make it difficult to implement effective vaccination programs [84]. Although the vaccine is available in developed countries, vaccination coverage has fallen sharply in some regions due to the population’s reluctance to be vaccinated. Consequently, eliminating measles requires a coordinated global response that includes improving vaccine coverage, eliminating vaccine hesitancy due to misinformation, and improving health infrastructure in developing countries. With greater collaboration between governments and health organizations, these obstacles can be overcome, and the global goal of measles elimination can be achieved in the near future.

## 3. Biological and Immunological Foundations

**FIGURE 2:**
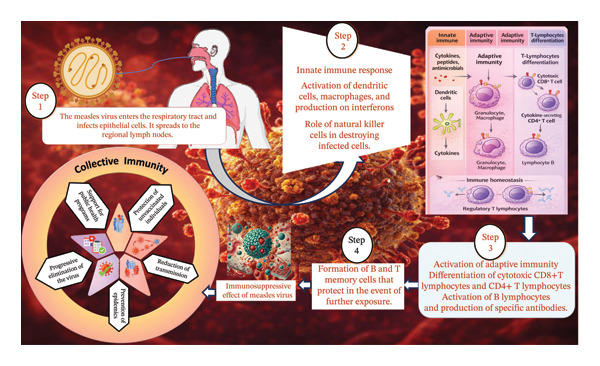
Mechanism of measles infection and immunity defenses (original figure created by authors).

The immune mechanisms of measles infection include several vital steps that begin with the pathophysiology of the measles virus, where the virus enters the body via the respiratory tract, replicates in mucosal cells, and leads to the spread of infection [85]. During the first phase, innate immunity plays a key role, with early defenses against viruses such as natural killer cells and interferons. Adaptive immunity is then activated by B cells, which produce antibodies, and T cells, which help destroy virus‐infected cells [86]. Over time, an immune memory is formed, providing long‐term protection against future infections [87]. However, measles can lead to immunosuppression, leaving the body vulnerable to other infections [88]. Herd immunity is therefore an important preventive strategy for limiting the spread of the disease, since the vaccination of the majority of individuals protects unvaccinated individuals (Figure 2).

### 3.1. Virology and Pathophysiology

Measles is a highly contagious viral disease transmitted by air; a sick person can infect an average of 18–20 people. The disease manifests itself as a rash with fever and other general signs, which can lead to serious complications and even death [32]. Measles is a highly contagious viral disease that first affects the respiratory system before spreading to other organs and can lead to serious complications and even death. It can occur at any age but is most common in children [33]. Measles is one of the most highly transmissible infectious diseases, and the risk of secondary attack exceeds 90% in susceptible individuals, meaning that an infected person can transmit the virus to nine out of ten unvaccinated close contacts. The virus is transmitted by air, via airborne droplets or direct contact with the nose or throat secretions of infected people. The virus remains active and transmissible in the air or on infected surfaces for up to 2 hours. It usually incubates for 7–14 days; in exceptional cases, up to 21 days in immunocompromised individuals. The period between exposure to the virus and the appearance of the rash is 14 days on average and can be as long as 21 days. The period of contagion begins 4 days before the appearance of the rash and ends 4 days afterward. Historically, measles appeared mainly in late winter and early spring in temperate regions [33]. After the incubation period, the first symptoms appear, usually lasting from 4 to 7 days and including high fever, cough, runny nose, and red, watery eyes. Koplik spots are small white patches on the inside of the cheeks, which may appear within two to 3 days of the onset of symptoms. The rash generally begins between the third and seventh day after the onset of symptoms; it is maculopapular in appearance (no vesicles), appearing first on the face and neck, rapidly spreading to the upper trunk and then to the arms and legs. It generally lasts 5–6 days before fading [33]. Measles can be serious for anyone who has contracted it, but certain groups are more likely to suffer complications from the disease: Children under 5, adults over 30, pregnant women, and people whose immune systems are weakened by the human immunodeficiency virus or other diseases. Measles also weakens the immune system, making sufferers, in general, and those in the above groups in particular, extremely vulnerable. Ear infections occur in around one in ten children with measles; diarrhea is reported in less than one in ten people with measles. Serious complications include respiratory including pneumonia, blindness, encephalitis, severe diarrhea, and dehydration, and, in pregnant women, premature delivery and low birth weight. Long‐term complications include subacute sclerosing panencephalitis, a very rare but fatal disease affecting the central nervous system of a person who has been infected with the measles virus 7–10 years previously, even if the person appears to have fully recovered [33].

The measles virus is a ribonucleic acid virus of the Paramyxoviridae family covered by a lipid envelope containing proteins that help it penetrate host cells. One of the key proteins on the virus surface is the hemagglutinin surface protein, which enables the virus to bind to the cluster of differentiation (CD) 46 and signaling lymphocytic activation molecule receptors on immune system cells, particularly T and B cells and glial cells. The virus is transmitted by respiratory droplets produced by coughing or sneezing. Once the virus enters the upper respiratory tract, it binds to these proteins, enabling it to enter host cells and begin replicating within them. The virus multiplies rapidly in infected tissue, spreading throughout the body and causing the characteristic symptoms of the disease [89, 90].

Measles is a highly contagious viral disease that mainly affects children but can also affect nonimmune adults. The measles virus is transmitted by respiratory droplets released when an infected person coughs or sneezes. Once inhaled, the virus infects the epithelial cells of the upper respiratory tract and then spreads to the regional lymph nodes, where it infects immune cells, notably macrophages and dendritic cells. These cells then transport the virus to secondary lymphoid organs, leading to systemic dissemination. Griffin reports that the measles virus infects cells expressing the CD150/signaling lymphocytic activation molecule family 1 protein, part of the CD2 family of transmembrane proteins. Signaling lymphocytic activation molecule family 1 is expressed on various immune cell types, such as immature thymocytes, memory B cells, and memory T cells. Its interaction with the measles virus leads to the fusion of the viral envelope with the cell membrane, enabling the cell to become infected. The virus replicates primarily in memory B and T cells, which can lead to a drop in lymphocytes in the blood, followed by immune proliferation and increased cell production by the thymus. In lymph nodes, measles rash is associated with increased numbers of B lymphocytes and continued production of measles virus–specific antibodies. These results highlight the mechanisms of measles virus infection and replication in immune cells and the consequences for the immune response [91]. Pidelaserra‐Martí and Engeland consider measles an oncolytic virus capable of specifically infecting and replicating in cancer cells, leading to their destruction. The measles virus vaccine shows tumor selectivity both during and after cell entry. Several cellular receptors, including CD150/signaling lymphocytic activation molecule, Nectin‐4, and the complement regulator CD46, are used by vaccine strains to enter cells. Although CD46 is present on all nucleated human cells, it must be overexpressed to enable efficient viral replication. Moreover, cancer cells often display defects in their innate antiviral immune response, making them more susceptible to viral replication. Vaccine strains of the measles virus are sensitive to interferon, while pathogenic strains produce proteins that antagonize interferon. In addition, the measles virus has a systemic antitumor effect, stimulating immune responses against tumors [92].

### 3.2. Innate Immunity and Early Antiviral Defenses Key Role

When the measles virus enters the body, it interacts with innate immunity as a first defense against infection. These defenses include macrophages that engulf the virus and present it to T cells to help activate adaptive immunity, natural killer cells that limit virus reproduction by destroying infected cells, and interferon molecules that help activate cellular defenses against viruses. Interferons enhance cells’ ability to fight viral infection and prevent viral replication in surrounding cells. These early responses slow the spread of the virus, but the ability of the virus to evade certain innate immune mechanisms means that an adaptive immune response is necessary to ensure longer‐lasting protection [93, 94]. Sørensen et al. highlight genetic factors influencing the immune response to the measles vaccine, notably by identifying genetic regions associated with plaque reduction neutralization test and immunoglobulin G antibody levels after measles–mumps–rubella vaccination. They claim that innate immune cells are activated upon infection with the measles virus. Dendritic cells and macrophages detect the virus via molecular pattern recognition receptors, such as Toll‐like receptors. Virus detection leads to the production of type 1 interferons (interferon‐α and interferon‐β), limiting viral replication and activating natural killer cells. Proinflammatory cytokines, such as interleukin‐6 and tumor necrosis factor‐α, are released to recruit more immune cells to the site of infection [95]. Ichimura et al. assert that as soon as the virus enters the body via the respiratory route, several immune mechanisms are triggered, including the activation of dendritic cells and macrophages, which capture the virus and present it to T lymphocytes; production of interferons *α* and β, antiviral cytokines that limit viral replication and promote activation of adaptive immunity; and activation of natural killer cells, which eliminate infected cells before specific lymphocytes are fully activated [96]. Suwoyo et al. estimate that when the measles virus enters the body, it initially targets cells of the respiratory system and infects immune cells such as dendritic cells and macrophages. Innate immune cells recognize the virus via Toll‐like receptors 3, 7, and 8, which detect viral ribonucleic acid. This detection leads to the activation of the nuclear factor‐kappa B pathway and production of pro‐inflammatory cytokines (interleukin 6, tumor necrosis factor‐α) and type I interferons (interferon‐α, interferon‐β), which limit viral replication and activate adaptive immune cells. Natural killer cells recognize infected cells and eliminate them by cytotoxicity, notably through the release of perforins and granzymes. However, the measles virus has developed evasion mechanisms, notably by reducing the expression of Class I major histocompatibility complex on infected cells, thus reducing their detection by natural killer cells [97]. Muñoz‐Alía et al. report that as soon as the virus enters the body, the innate immune system is activated. Infected cells recognize the measles virus via pattern recognition receptors, such as Toll‐like receptors, leading to the production of proinflammatory cytokines and type I interferons. These molecules signal the presence of infection and initiate an antiviral response by inducing the expression of interferon‐stimulated genes that inhibit viral replication [98]. Yang et al. asserted that upon infection, the measles virus is recognized by pattern recognition receptors present on innate immunity cells, such as dendritic cells and macrophages. This recognition leads to the production of proinflammatory cytokines and the activation of antiviral mechanisms. However, the measles virus has developed strategies to evade this initial response, notably by inhibiting the production of interferons, enabling it to replicate efficiently [99]. Liu et al. highlight the immune mechanisms involved in measles protection and infection, focusing mainly on the evolution of immunity induced by the measles–mumps–rubella vaccine in children aged 3 to 7. The measles–mumps–rubella vaccine primarily stimulates the production of immunoglobulin G antibodies to measles, mumps, and rubella. Liu et al. show a progressive decline in antibody levels, particularly against mumps, underlining the importance of immune memory. Following the administration of a dose of measles–mumps–rubella vaccine, children develop a robust humoral response characterized by high levels of immunoglobulin G. Peak immunoglobulin G titers generally occur in the months following vaccination. Over time, a decrease in geometric mean antibody levels is observed, suggesting a progressive decline in protection. Liu et al. highlight that mumps seropositivity decreased from 79.6% in 2015 to 71.4% in 2018, which is below the threshold required for herd immunity (88%–92%). The phenomenon of negative serological conversion is observed, meaning that some initially seropositive children become seronegative, increasing their susceptibility to infection. This negative conversion is more pronounced in younger children, indicating a decrease in their susceptibility to infection. Liu et al. reveal that 6.1%–7.4% of children between 2015 and 2016 and 9.0%–9.9% between 2016 and 2018 were probably exposed to asymptomatic infections. These infections may act as a natural immune boost, explaining why some children maintain a certain level of antibodies despite the general decline in geometric mean levels [100]. Haralambieva et al. assert that the measles virus uses the signaling lymphocytic activation molecule (CD150) receptors, expressed on immune system cells (dendritic cells, macrophages, activated T and B cells), as well as CD46, present on the majority of human cells, as an entry point, and that Nectin‐4 is another receptor enabling infection of epithelial cells, thus facilitating dissemination of the virus throughout the body, and that the recognition of the virus by Toll‐like receptors and other pathogen motif sensors such as CD209 triggers immune signaling. They also report that signaling pathways activate the production of proinflammatory cytokines, including interferon‐α, which inhibits viral replication and activates immune cells, and interleukin‐6 and tumor necrosis factor *α*, which participate in the inflammatory response and recruitment of immune cells [101].

### 3.3. Activation of Adaptive Immunity: B and T Cells in Viral Clearance

Once innate immunity has been activated, the adaptive immune system steps in to increase the effectiveness of defenses against the virus. This response includes CD4+ T helper cells, which recognize the virus via antigen‐presenting cells and help activate CD8+ killer T cells, which attack and destroy infected cells, and B lymphocytes, which produce measles virus antibodies that bind to viral proteins and prevent the virus from binding to healthy cells, thus preventing it from entering host cells. The combined activation of these cells destroys the virus and rids the body of infection [102]. Adaptive immunity is essential for clearing the virus and establishing lasting protection. B lymphocytes produce immunoglobulin M antibodies (early response) and immunoglobulin G antibodies (prolonged response). Sørensen et al. demonstrated an association between antimeasles immunoglobin G levels and certain genetic variations, notably human leukocyte antigen‐B1801, which was negatively correlated with immunoglobulin G production [95]. Plaque reduction neutralization test neutralizing antibodies play a key role in protecting against reinfection by preventing the virus from entering host cells. For the cellular response, T‐cell–mediated immunity, cytotoxic CD8+ T lymphocytes recognize and destroy infected cells, and CD4+ (helper) T lymphocytes secrete cytokines to help activate B and CD8+ lymphocytes. Sørensen et al. identified a single‐nucleotide polymorphism (rs3005891) influencing the FYN binding protein 2 gene, involved in T cell receptor signaling, suggesting a variation in the cellular response [95]. Ichimura et al. consider that the adaptive immune response is crucial for virus elimination and the development of a lasting immune memory. Activated B lymphocytes produce specific immunoglobulin M antibodies initially, followed by immunoglobulin G in the long term. Measles immunoglobin M can be detected as early as 3–5 days after the appearance of the rash and persists for several weeks. Antimeasles immunoglobin G appears later and persists for life, conferring long‐lasting protection. Cytotoxic CD8+ lymphocytes eliminate infected cells by cell lysis. CD4+ lymphocytes help mature B lymphocytes and produce protective antibodies. A key aspect of measles is that it induces a state of transient immunosuppression by affecting immune memory, making the host more vulnerable to secondary infections for several weeks to months after infection. In addition, they point out that the measles vaccine uses a live attenuated virus that stimulates the immune response without causing disease. It is administered as a combined measles–rubella or measles–mumps–rubella vaccine. Ichimura et al. hypothesize that the postvaccination immune response manifests itself in the production of specific immunoglobin G, which confers long‐term protection, activation of memory B lymphocytes, enabling a rapid response in the event of subsequent exposure to the virus, and induction of memory CD4+ and CD8+ T lymphocytes, which reinforce cellular immunity. Also, Ichimura et al. show that the prevalence of antimeasles IgG was 62.6%, well below the 95% threshold required to interrupt virus transmission, that without boosters or enhanced routine immunization, the effect of vaccination campaigns gradually diminishes, and that vaccine preservation (cold chain), storage conditions and the test kits used can influence the immune response measured [96]. Suwoyo et al. report that, following infection, antigen‐presenting cells migrate to the lymph nodes, where they present viral peptides to T cells via the Class I major histocompatibility complex and Class II major histocompatibility complex. Cytotoxic CD8^+^ T cells recognize infected cells and destroy them via the release of granzyme B and perforin. This response is essential for eliminating infected cells and limiting the spread of the virus. CD4^+^ T cells help produce antibodies by activating B cells. They also orchestrate the inflammatory response via the production of cytokines, helping to amplify the immune response. B lymphocytes play a key role in the production of measles virus‐specific antibodies. Immunoglobulin M is the first antibody produced during acute infection. It neutralizes the virus and activates the complement cascade, facilitating the elimination of viral particles. Suwoyo et al. show that antisevere acute respiratory syndrome coronavirus 2 immunoglobin M decreases rapidly after vaccine exposure, as is also observed with measles. Immunoglobin G takes over, providing lasting protection. After natural infection or vaccination, immunoglobin G levels persist at high levels throughout life, conferring long‐lasting immunity against measles. Suwoyo et al. suggest that immunoglobin G may interact with other antigens, explaining a possible cross‐reactivity with severe acute respiratory syndrome coronavirus 2 after measles vaccination [97]. Muñoz‐Alía et al. suggest that the adaptive response is crucial for eliminating the virus and establishing a lasting immune memory. Dendritic cells present viral antigens to T lymphocytes in the lymph nodes. Cytotoxic CD8+ T cells recognize and destroy infected cells, while CD4+ T cells help activate B cells. The latter differentiate into plasma cells, which produce specific antibodies against the measles virus, neutralizing the free virus and preventing further cellular infection [98]. Yang et al. report that the activation of adaptive immunity involves the presentation of viral antigens to T cells by antigen‐presenting cells. CD4+ T helper cells orchestrate the immune response by activating B lymphocytes, which differentiate into MeV‐specific antibody‐producing plasma cells. At the same time, CD8+ cytotoxic T lymphocytes eliminate infected cells [99]. Haralambieva et al. argue that the humoral response is largely dependent on antigen presentation by dendritic cells to CD4+ helper T cells, which in turn activate antibody‐producing B cells. The major histocompatibility complex modulates antigen recognition and presentation to immune cells. Genetic polymorphisms in non‐HLA genes, such as those involved in interleukin 2, 10, 12β, 4Rα, 12Rβ1, and 7R cytokine signaling, are associated with interindividual differences in antibody production. The CD46 gene polymorphism (single‐nucleotide polymorphism rs2724384) is strongly correlated with variations in IgG antibody titer and differences in the secretion of interferon‐α, interleukin 6, and tumor necrosis factor‐α. A strong Th1 helper T cell response via interferon‐γ is generally associated with effective protection against measles virus. Certain polymorphisms in the signaling lymphocytic activation molecule receptor genes (single‐nucleotide polymorphism rs164288) influence antibody levels and the cellular immune response [101].

### 3.4. Immune Memory and Long‐Term Protection

One of the most important aspects of the immune response to measles is immunological memory. After recovery from infection, immune memory preserves the T and B cells that can recognize the virus in the future. This memory enables the body to provide a rapid and effective response against measles if it is exposed to the virus again. This is the basis of measles vaccines, which are among the most effective ways of preventing the disease and ensuring long‐term protection [91].

### 3.5. Measles‐Induced Immunosuppression

Although immunity to measles is effective, the virus causes the immune system to become less able to fight other diseases after infection. The measles virus disrupts important immune cells such as T lymphocytes, reducing the body’s ability to fight infection. The measles virus causes imbalances in immune memory, increasing the chances of other diseases infecting the body. After contracting measles, patients face increased susceptibility to illnesses such as pneumonia and intestinal infections due to a weakened immune system. These effects lead to increased morbidity and mortality rates, particularly in infants and children who have not received the vaccine, underlining the importance of vaccination as a means of preventing this virus and its devastating immunological effects [103]. The measles virus not only affects the respiratory system but also causes severe immune system disorders that can persist long after the infection has healed. Preventing measles through vaccination, therefore, remains the most effective way to protect against this dangerous disease and its negative effects on the immune system. Park et al. state that the measles virus is a highly contagious, negative‐polarity, single‐stranded ribonucleic acid paramyxovirus that primarily infects epithelial cells and lymphocytes. When it enters the body, it crosses the epithelial barriers of the respiratory tract to reach immune cells, notably macrophages and dendritic cells. These cells act as initial reservoirs, allowing the virus to replicate and spread to regional lymph nodes. Once in the lymphatic system, the measles virus infects B and T lymphocytes, causing transient but profound immunosuppression. This suppression weakens the host’s immune response, increasing susceptibility to further infections. Paradoxically, despite this immunosuppression, measles virus infection induces a robust adaptive immune response, characterized by the production of specific neutralizing antibodies and the development of memory T cells, conferring long‐term immunity against measles. The measles virus also has the ability to induce autophagy, a lysosomal degradation process involved in both innate and adaptive immunity. Some attenuated strains of the virus can trigger successive waves of autophagy in infected cells, which can influence the viral cycle and the host immune response. Measles vaccination uses a live, attenuated strain of the measles virus, which stimulates the immune system without causing disease. This vaccination induces a humoral immune response, with the production of specific antibodies, and a cellular response, with the activation of T lymphocytes. These mechanisms provide long‐lasting protection against measles [104]. Consequently, measles virus infection involves a complex interplay between innate and adaptive immunity. The virus uses strategies to modulate the host’s immune response, but the organism generally develops robust and long‐lasting immunity after infection or vaccination. Suwoyo et al. suggest that the measles virus possesses mechanisms that suppress the immune response. It decreases the production of type I interferons, thereby reducing immune alertness. It induces transient immunosuppression after infection, increasing the risk of other opportunistic infections. They add that the measles vaccine is a live attenuated vaccine that mimics natural infection without causing severe disease. It triggers rapid, massive production of protective Immunoglobin G, an immune memory provided by memory B and T lymphocytes, guaranteeing long‐term protection [97]. Laksono et al. suggest that the measles virus infects mainly cells expressing the CD150 (signaling lymphocytic activation molecule) receptor, a marker found on immune system cells, including dendritic cells, memory T and B lymphocytes, and natural killer cells. This infection leads to transient lymphopenia, i.e., a reduction in the number of circulating lymphocytes, which weakens the immune response. One of the most striking aspects of measles infection is the destruction of memory lymphocytes, leading to a state of immune amnesia. Laksono et al. observed a significant reduction in memory T and B lymphocytes after infection, observable more than a month after recovery, a loss of pre‐existing antibodies due to infection, the depletion of long‐lived plasma cells in the bone marrow, and a decrease in memory B cell clones, which are not reconstituted after infection. They also claim that measles virus infection alters the composition of the lymphocyte compartment through an increase in transitional B cells, which are immature lymphocytes emerging from the bone marrow with reduced proliferative capacity, and an increase in regulatory T cells, which inhibit the immune response, thus contributing to prolonged immune suppression. Laksono et al. also demonstrate that measles vaccination effectively protects against infection and its immunosuppressive effects, as attenuated virus vaccines do not induce systemic viral replication or viremia. Unlike natural infection, vaccination does not induce immune amnesia or alter the antibody repertoire. In nonhuman primates vaccinated with a recombinant strain of measles virus expressing a fluorescent protein, no immunosuppressive effects were observed after experimental wild‐type infection [105].

### 3.6. Importance of Herd Immunity and Control Strategies

Herd immunity is an essential method of combating epidemic diseases, including the measles virus, and occurs when the number of people immunized against a disease reaches a certain threshold within a community, preventing the disease from spreading widely. It is reached when enough people are immunized naturally or by vaccination, thus preventing the virus from circulating. Measles requires a high vaccination coverage of 95% or more to interrupt transmission, limit the spread of the disease, and protect those who cannot receive the vaccine, such as infants or immunocompromised people. Ichimura et al. recommend increasing routine vaccination coverage through better awareness and accessibility, organizing targeted vaccination campaigns with effective follow‐up to avoid immune decline, and improving epidemiological surveillance systems to identify at‐risk populations [96]. Haralambieva et al. suggest that the vaccinomics approach, which integrates genomics, immunogenetics, and functional studies, provides a better understanding and prediction of vaccine response, advanced technologies such as transcriptomic and proteomic analyses, as well as bioinformatics models, help identify genetic markers of vaccine protection, epitope‐prediction, and structural modeling algorithms could improve the design of future vaccines [101]. In addition to vaccines, awareness campaigns and increased access to health services are effective strategies for combating epidemic measles. Maintaining good hygiene practices and strengthening collaboration between governments and health organizations can help reduce the spread of the virus and boost community resistance.

## 4. Effects of Measles on Society, Environment, Health, and Smart Technologies

Measles is a highly contagious viral disease with multiple impacts not only on public health but also on the environment, biodiversity, the education system, and the psychology of children and parents, with differences between past, present, and future, as well as between developed, developing, and underdeveloped countries [106–108].

For the impact of measles on the environment and biodiversity, in the past, measles has had no direct environmental impact, but epidemics have led to population declines in certain regions, perhaps giving ecosystems a chance to recover temporarily from human activity [109]. In some areas, epidemics may have led to a decline in agricultural and hunting activities, allowing wildlife to thrive for short periods. At present, the direct environmental impact of measles is limited, but vaccination campaigns can affect the environment through medical waste (such as needles and syringes). Increased global mobility favors the spread of disease, which risks depleting health and economic resources that could otherwise be used to preserve the environment. In the future, if global vaccination programs continue, measles epidemics are likely to be reduced, which will reduce indirect environmental impacts [110]. If epidemics recur due to inadequate vaccination campaigns or vaccine resistance, this can have health and economic consequences that affect investments in environmental conservation. Developed countries have robust healthcare systems that reduce the environmental impact of epidemics. In developing and underdeveloped countries, epidemics can weaken health and economic resources, reducing interest in biodiversity conservation. In underdeveloped countries (with ongoing unrest), epidemics can increase the burden on health and economic services, further neglecting the environment [111]. Environmental and climatic changes are increasingly recognized as modifiers of infectious disease transmission dynamics, potentially influencing outbreak patterns and geographic spread [112]. Climate‐related disruptions have already amplified infectious disease outbreaks in Africa, such as cholera, illustrating the vulnerability of health systems to compounding crises [113].

For the impact of measles on the education system: In the past, measles epidemics forced children to miss school for long periods, affecting their academic performance [114]. In some cases, the disease has led to deaths among children, reducing the number of pupils enrolled in schools. Currently, in developed countries, measles is controlled by vaccination, reducing its impact on education. In developing countries, measles epidemics continue to cause children who are ill or caring for infected family members to miss school. In some poor areas, measles epidemics can lead to the temporary closure of schools to prevent the spread of infection. In the future, if vaccination campaigns continue, the impact of measles on education should be minimal. If vaccine resistance spreads, we could see new epidemics affecting the educational process. Countries with fragile health systems could face educational crises linked to the epidemic [115]. For the impact of measles on the psychology of children and parents: In the past, children were extremely afraid of the disease because of its prevalence and the lack of effective treatment [116]. Measles deaths were deeply traumatic for parents. There was greater susceptibility to epidemics due to a lack of medical awareness. Today, in developed countries, measles is less of a concern because vaccines are available. In developing countries, an epidemic can cause psychological stress for families, especially in the absence of health services. Parents who oppose vaccination may feel guilty if their children fall ill. In the future, if vaccination campaigns continue, measles‐related psychological anxiety should decrease. If vaccine resistance increases, fear of the disease may return, affecting the mental health of children and parents alike [72]. As a result, in developed countries, the impact of measles has been limited by vaccines, but vaccine resistance could pose a risk in the future. In developing and underdeveloped countries, measles remains a health and social challenge, affecting education, mental health, and the environment (Table 2). The global fight against measles through vaccination and health education is essential to reduce its impact in the future.

**TABLE 2:** The difference between the past, the present, and the future in the impact of measles.

Scope	Past	Present	Future	References
Environment	Indirect effect through reduction in human activity	Limited impact via medical waste and changes in the use of healthcare resources	Depending on the vaccination, the environmental impact may be minimal or aggravated by the epidemic.	[36]
Biodiversity	Some temporary improvement due to a reduction in human activity	Minimal impact except in areas economically affected by epidemics	Biodiversity could be affected if environmental funding is weakened by pandemics.	[117]
Education system	Truancy and low completion rates	Limited impact in developed countries, significant impact in developing and underdeveloped countries. Depending on the success of vaccination campaigns, the situation could improve or worsen.	Depending on the success of vaccination campaigns, the situation could improve or worsen.	[118]
Child and parent psychology	Constant fear of illness and profound psychological effects	Concern varies according to the level of vaccination coverage	If vaccination rates fall, psychological stress can increase.	[119]

The measles epidemic has also had an impact on the development of advanced technologies. In the past, financial and human resources have been diverted to fighting epidemics, reducing funding for research and development in smart technologies [120].

Recent advances in digital epidemiology have introduced data‐driven tools that may complement traditional measles surveillance systems [121]. Machine learning–based models have been explored to estimate outbreak risk by integrating vaccination coverage data, population density, mobility patterns, and historical incidence records. These models typically generate district‐level risk maps or short‐term outbreak probability estimates, which can support the prioritization of supplementary immunization activities. In addition, anomaly detection algorithms applied to syndromic surveillance databases may assist in identifying unusual clusters of febrile rash illnesses before laboratory confirmation [122]. However, most of these approaches remain at pilot or research stages, and their predictive performance depends heavily on data completeness, reporting quality, and interoperability of health information systems. In low‐ and middle‐income countries, implementation challenges include fragmented digital infrastructure, limited trained personnel, maintenance costs, and concerns related to data governance, privacy, and algorithmic bias [123–125]. Therefore, artificial intelligence should be considered a complementary decision‐support tool rather than a substitute for robust field epidemiology and vaccination programs.

## 5. Interventions to Control and Eliminate Measles

### 5.1. Measles Epidemiological Surveillance System: Methodology, Case Definition, and Confirmation Criteria

A comprehensive epidemiological surveillance system was set up in 2010, based on biological confirmation and epidemiological investigation of all suspected cases of measles. This surveillance system rapidly achieved the performance criteria required by the World Health Organization, which classified our country in the pre‐elimination phase [32]. In epidemic zones, active surveillance is conducted in which healthcare professionals visit facilities to look for cases, with epidemiological investigation of each case detected. In nonepidemic areas, semiactive surveillance, in which healthcare professionals contact facilities to check for cases, with epidemiological and virological investigation of each case detected, and genomic surveillance, which involves analysis of the virus’s genetic material to identify its variants, trace its spread, and assess the effectiveness of control measures [33]. A suspected case is any person presenting with a nonvesicular maculopapular rash, in a febrile context, with or without cough, rhinitis, or conjunctivitis. All suspected cases are investigated epidemiologically and/or biologically, depending on whether the area is epidemic or not. A confirmed case is any suspected case confirmed positive in the laboratory, by the detection of immunoglobin M or change in immunoglobin G titer, or the detection of viral ribonucleic acid by RT‐PCR. A case confirmed by epidemiological link is any suspect case that has not been confirmed by a laboratory, but for which a spatial and temporal link (period of contagiousness) has been demonstrated with a laboratory‐confirmed case or another case of measles confirmed by epidemiological link. A measles‐related death is any death occurring within 30 days of the onset of rash in a clinically, laboratory, or epidemiologically confirmed case of measles, related to a measles complication and not attributable to another cause [33].

**FIGURE 3:**
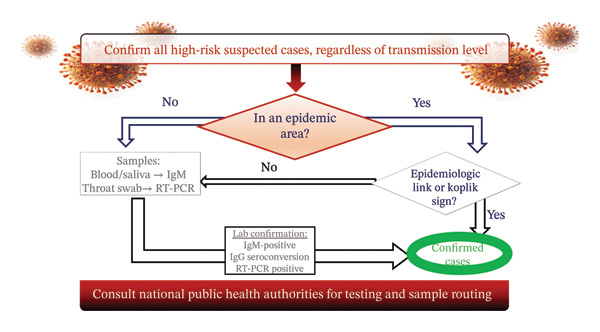
Diagnostic confirmation flowchart for a suspect case of measles (original figure created by authors).

The flowchart in Figure 3 describes the process for confirming a suspected case of measles, based on the epidemiological situation and the results of biological tests. It is based on a structured decision‐making approach, combining epidemiological, clinical, and biological criteria. The starting point for the diagnostic process is a suspected case of measles, i.e., a patient presenting symptoms suggestive of the disease (febrile exanthem, cough, conjunctivitis, etc.). The first decision is based on the presence or absence of an epidemic zone: If the patient is in an epidemic zone, the analysis is based on epidemiological and clinical criteria. If the patient is not in an epidemic zone, virological or serological diagnosis is required. If the patient presents a clear epidemiological lineage (contact with a confirmed case) or the Koplik sign (whitish patches inside the cheeks, pathognomonic of measles), then the case is directly confirmed. If none of these criteria is observed, biological tests are required. Outside epidemic zones, a blood or saliva sample is taken to detect measles‐specific IgM antibodies. A throat, nasopharyngeal, or salivary swab is taken to test for viral RNA by RT‐PCR. A case is biologically confirmed if antimeasles immunoglobin M is detected, seroconversion is observed with an increase in the titer of specific immunoglobin G, and/or viral RNA is detected by RT‐PCR. Epidemiological lineage is an important criterion during an epidemic. Contact with a confirmed case and typical symptoms are sufficient to establish a diagnosis. The Koplik sign is a specific but transient clinical marker, generally present 1–2 days before eruption. Measles immunoglobulin M tests confirm recent infection, as these antibodies appear 3–5 days after the rash and persist for around 1 month. The detection of immunoglobin G and its increase in two spaced samples (seroconversion) indicates an ongoing or recent infection. RT‐PCR enables the direct detection of measles viral RNA, making it a sensitive and specific technique. Nasopharyngeal, salivary, and sometimes urinary swabs allow better detection of the virus, especially in the early stages of the disease.

### 5.2. Biological Diagnosis of Measles

Biological diagnosis of measles is based on the detection of specific immunoglobin M antibodies or a fourfold increase in immunoglobin G titer on two samples taken 10–20 days apart, and the detection of viral RNA by RT‐PCR 5 days before and 8–12 days after the onset of the rash. Specific IgM antibodies appear around the same time as the rash, peaking during the first week after onset, and are rarely detected after 6–8 weeks [126]. A single blood or saliva sample for IgM detection is generally sufficient to make the diagnosis, as it is most often positive if taken between the 3rd and 28th days following the onset of the rash [127]. IgM can be detected in both blood and saliva samples, using the same kinetics. Saliva samples are collected by rubbing the inside of the patient’s cheeks and gums for about a minute, using the small sponge supplied in the kit, until it is soaked in saliva. However, during the first 3 days of the rash, PCR is indicated. IgG measles antibodies are generally produced and detectable a few days after IgM. The timing of the IgM and IgG response varies from individual to individual, but IgG should be detectable 7–10 days after the onset of the rash, peaking about 2 weeks later, and persisting for life [128]. PCR on a nasopharyngeal, throat, saliva, or urine sample is positive a few days before the appearance of the rash and up to 8–12 days afterward; beyond this time, PCR loses its interest. It should be noted that PCR is also used to determine whether or not the sample can be genotyped by sequencing [33].

### 5.3. Innovations in Measle Vaccination

**FIGURE 4:**
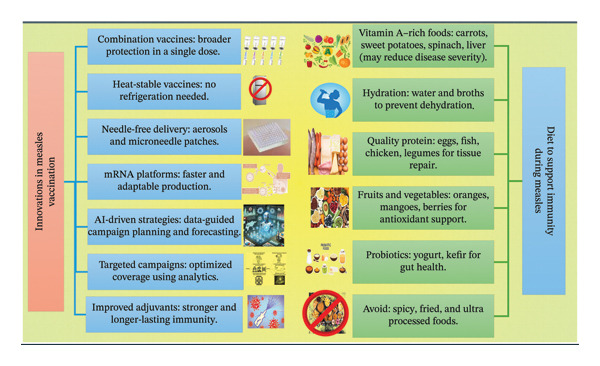
Innovative measles vaccination and immune‐boosting diets for treatment (original figure created by authors).

There is no specific treatment for measles; care consists essentially of relieving symptoms, preserving the patient’s comfort and preventing complications (Figure 4). It is important to eat well and drink enough water, and rehydration will be necessary to replace any fluid losses caused by vomiting and diarrhea. Doctors can use antibiotics to treat pneumonia, and ear and eye infections. All children and adults with measles should receive two doses of vitamin A supplements, administered 24 h apart. This treatment restores the low levels of vitamin A found even in well‐nourished children and can help prevent eye damage and blindness. Vitamin A supplementation also reduces the number of deaths caused by measles [33].

Vaccination is the most effective way to prevent measles; the vaccine is safe and highly effective. Children should receive two doses of the vaccine to be immunized: Two doses of measles vaccine are around 97%–100% effective in preventing the disease in the event of exposure to the virus, while a single dose is around 93% effective. In Morocco, the measles vaccine is combined with the rubella vaccine as part of the National Immunization Program, which is provided free of charge at all primary healthcare facilities. The first dose is administered at 9 months of age and the second at 18 months. Vaccine‐induced antibodies generally appear only a few days after vaccination, and vaccinated individuals are usually fully protected after around 2–3 weeks. Due to the extremely high infectivity of measles, vaccination coverage must be at least 95% to avoid the spread of the virus in the community [33].

It is important to distinguish between strategies currently adopted for measles prevention and innovations that are still in clinical research or evaluation. The internationally proven approach is based on a two‐dose schedule of measles, supplementary immunization activities, and catch‐up campaigns aimed at raising coverage, effectively reducing morbidity, mortality, and outbreaks. In contrast, some modern approaches, such as inhaled vaccines or technology‐based vaccines, are mRNA, a promising trend aimed at improving ease of administration or enhancing the immune response, but it is still in the development or evaluation stages and has not yet been adopted in national measles immunization programs. Therefore, while it is important to follow up on these research innovations, the current control of measles depends mainly on the rigorous application of approved strategies and the increase in coverage with routine and complementary vaccination.

Vaccination against measles was introduced in Morocco in 1982 as part of the Expanded Immunization Program, followed in 1986 by the National Immunization Program, which involved the administration of a dose of measles vaccine at 9 months of age. Nationwide vaccination coverage had reached and even exceeded 90% by 1995, with a marked improvement in measles‐related morbidity and mortality, but without preventing the cyclical occurrence of epidemic outbreaks. The National Immunization Program has opted for combined measles–rubella vaccination and for the administration of a second dose at the age of 18 months, starting in 2014. Two mass vaccination campaigns were organized in 2008 and 2013 [32]. In nonepidemic areas, vaccinate selectively and catch up with measles–rubella vaccine and other vaccines according to the national vaccination schedule and the child’s age. In epidemic areas, rapidly organize a mass nonselective measles–rubella vaccination campaign starting at 6 months of age. In closed communities, vaccinate all residents, including staff, regardless of vaccination status and measles history. However, the contraindications to measles–rubella vaccination must be taken into account (measles–rubella vaccine should not be administered to pregnant women, immunocompromised individuals, or anyone with a sensitivity to measles–rubella vaccine following the administration of a first dose) [33]. It is not recommended to revaccinate people who have received a dose of measles–rubella vaccine in less than 30 days, regardless of their age. In epidemic zones or in the event of contact with a case of measles, the measles–rubella vaccine can be administered from the age of 6 months. Infants aged between 6 and 8 months should receive one dose of the measles–rubella vaccine. They will then be vaccinated with two doses (at 9 and 18 months) according to the vaccine schedule. Infants aged 9 months and above who have received their first dose of vaccine should receive their second dose 4 weeks apart, without waiting until they are 18 months old. Infants aged 9 months and above who have never been vaccinated, or whose vaccination status is unknown, should receive two doses of measles–rubella vaccine, 4 weeks apart. Measles–rubella vaccine should not be administered to pregnant women, people who have developed anaphylactic shock following a previous dose of measles–rubella vaccine, and immunocompromised people: human immunodeficiency virus, hereditary deficiencies, chemotherapy, biotherapy, cancer, and aplasia. When measles–rubella vaccine is administered to women of childbearing age, they should be advised to take precautions against pregnancy for 1 month. Women inadvertently vaccinated with measles–rubella vaccine during pregnancy can be reassured immediately. Such an incident would not be a reason to recommend the termination of the pregnancy. Breast‐feeding is not a contraindication to measles–rubella vaccination, which can be administered to breastfeeding mothers without any risk to their babies. There is no evidence of measles vaccine virus in breast milk [33].

### 5.4. Supportive Nutritional Care in the Clinical Management of Measles

Measles is a viral disease that can lead to severe complications, particularly among malnourished children. While vaccination remains the primary and most effective strategy for preventing measles and controlling its spread, supportive care plays an important role in clinical management. This includes attention to nutritional status, especially in settings where malnutrition is prevalent.

Aurangzeb et al. asserted that encephalitis, nonvaccination, and undernutrition were significantly associated with mortality in children with complications of measles at the Children Hospital, Pakistan Institute of Medical Sciences, Islamabad, Pakistan, between 2013 and 2017 [129]. The World Health Organization recommends vitamin A supplementation for all children diagnosed with measles in areas where vitamin A deficiency is prevalent. Clinical evidence demonstrates that vitamin A reduces measles‐related mortality and complications, including ocular damage and pneumonia. Vitamin A administration should be considered a therapeutic supportive intervention during acute infection and not a substitute for vaccination. Benn et al. assumed that neonatal vitamin A supplementation may interact with vaccines given several months later for Guinea‐Bissau between 2002 and 2008 and that this may have implications for the planning of future child intervention programs [130]. Jensen et al. examined the immunological effects of vitamin A supplementation administered with measles vaccine. The World Health Organization recommends this supplementation after 6 months, and many children receive it with the vaccine [131]. Jensen et al. showed that vitamin A supplementation influences the immune system differentially according to gender and previous exposure to vitamin A supplementation [131]. Vitamin A supplementation is associated with an increase in C‐reactive protein ≥ 5 mg/L, an inflammatory marker, in 28% of children versus 12% on placebo [131]. In boys, vitamin A supplementation reduces leukocytes, lymphocytes, monocytes, and basophils, whereas it increases them in girls [131]. In girls who had already received vitamin A supplementation, it stimulated proinflammatory and Th1 responses, whereas it reduced them in those who had never been supplemented. The opposite effect was observed in boys [131]. As a result, vitamin A supplementation administered with measles vaccine has complex immunological effects, varying according to gender and history of vitamin A supplementation. Kumari and Kutti show that there is a significant association between a negative immune response to measles vaccine and undernutrition. Anemia is also a factor associated with a less effective immune response, suggesting that nutritional deficiencies may affect the body’s ability to respond to vaccination. A lack of exclusive breastfeeding up to 5 months is associated with a weaker immune response, underlining the importance of infant feeding in the early months to support the vaccine response [132]. Martins et al. assessed the impact of measles vaccination on hospitalization rates among children in a Guinea‐Bissau population between 2003 and 2007. Children were randomly assigned to receive measles vaccine at 4.5 months or not before all were vaccinated at 9 months. They found that the measles vaccine group had a lower rate of hospitalization than the unvaccinated group, with a particularly marked reduction in girls and for respiratory infections. The results suggest that early measles vaccination could have beneficial effects on child health, reducing hospital admissions and healthcare costs, particularly for measles and respiratory infections [133]. Ni et al. explore the relationship between infants’ body vitamin A nutritional status, hepatitis B antibody level, and measles immunoglobulin G antibody concentration by collecting 2 mL of infants’ venous blood in accordance with inclusion criteria, from vaccination agencies in Shandong province, China. In parallel, a combined method of reviewing 24‐h food records and 2‐day food diaries of the infants’ caregivers was used to learn about the food ingested by the infants and its quantities over the successive 72 h. Their results suggest that vitamin A in serum may be linked to the maintenance of effective levels of antihepatitis B antibodies and immunoglobulin G antibodies against measles [134]. However, Shimelis et al. reported that a public health campaign in Sierra Leone, which integrated vitamin A supplementation and measles vaccination for children aged 6–59 months, resulted in fever and pain at the injection site and postvaccination adverse events [135]. Malnutrition, including wasting and anemia, has been associated with more severe measles outcomes and weaker immune responses. The bidirectional relationship between infection and malnutrition may create a cycle of worsening health status. Therefore, appropriate treatment of acute malnutrition forms part of comprehensive supportive care for affected children. Noori et al. asserted that mathematical modeling suggests that integrating malnutrition treatment programs with improved vaccination coverage may result in greater reductions in measles incidence and mortality compared with implementing either intervention alone. These findings highlight the complementary role of nutritional interventions alongside immunization programs, without diminishing the central role of vaccination in prevention [136]. Sulaiman et al. confirm that the measles epidemic in the mining areas of Abu Hamad, Sudan, had serious consequences, attributed to poor environmental conditions, overcrowding, poor nutrition, and lack of vaccination [137]. Proteins are very important for regenerating damaged tissue and strengthening the body’s ability to fight infection [138].

In some contexts, vitamin A supplementation campaigns are integrated with measles vaccination programs to improve access to high‐risk populations. Reported adverse events are consistent with the established safety profiles of vaccines and do not alter routine immunization recommendations.

It is important to emphasize that nutritional interventions provide supportive benefits in clinical management and overall child health, but do not provide specific protection against measles virus infection. High coverage with two doses of measles‐containing vaccine remains the cornerstone of measles prevention and control.

### 5.5. Sustainable Strategies for Measles Elimination

India’s recent measles outbreaks demonstrate how temporary disruptions in immunization services can threaten national elimination targets, particularly when herd immunity thresholds (> 95%) are not maintained [139]. The study conducted in Tianjin, China, reveals that measles immunity remains insufficient to reach the 95% herd immunity threshold, despite high vaccination coverage among young people. The proportion of nonimmunized individuals is particularly high among adults aged 20 to 39, with less than 90% protection in this age group. Infants rapidly lose their passive immunity before the age of vaccination (8 months), but vaccination subsequently increases immunity in young children. However, current vaccination coverage is not sufficient to compensate for the lack of immunization among adults, thus compromising the goal of eliminating measles in China [53].

The 2018–2019 measles epidemic in Israel mainly affected the Jerusalem district, with a high incidence rate (176 per 10,000 inhabitants). The majority of cases (75.5%) involved children under the age of 15, nearly half of whom were under the age of 5. Most of those infected (81.1%) were unvaccinated, including ineligible infants and children who had not been vaccinated despite being eligible. The study analyzed 2254 cases, of which 31.8% were laboratory‐confirmed and 68.2% epidemiologically linked. Factors associated with laboratory confirmation included the time of illness onset, the presence of additional cases in the household, the place of treatment, and vaccination status. This epidemic underlines the importance of strengthening vaccination coverage and epidemiological surveillance to achieve measles elimination [140].

The spring 2017 measles outbreak in Minnesota primarily affected the undervaccinated Somali community, requiring rapid intervention by public health authorities. The outbreak was well advanced when the first cases were confirmed, leading to immediate containment measures, including testing, epidemiological investigation, accelerated vaccination, and temporary exclusion of unprotected exposed individuals. A total of 75 cases were recorded: 91% of them in unvaccinated individuals, and 28% requiring hospitalization. The majority of transmissions occurred in day‐care centers and homes, with an attack rate of 91% among unvaccinated household contacts. Over 51,000 doses of measles–mumps–rubella vaccine were administered, and $2.3 million was spent to contain the epidemic. This crisis highlights the importance of rapid health response, vaccination, and strong collaboration between public health authorities and affected communities to prevent and control measles [141].

Vaccines are among the safest medical products, with rigorous testing and continuous monitoring. Although adverse events may coincide temporally with vaccination, deaths directly caused by vaccines are extremely rare. Scientific studies have found no evidence of a widespread link between vaccination and death, except in exceptional cases, such as anaphylaxis, systemic infection in immunocompromised people after a live vaccine, or certain rare complications linked to specific vaccines. During the 2014–2015 measles epidemic in the United States, unfounded rumors of measles–mumps–rubella vaccine‐related deaths circulated online, necessitating a response from public health authorities. It is scientifically invalid to draw conclusions about a link between vaccines and deaths solely from spontaneous reports or anecdotes, without thorough analysis of epidemiological data [142].

A study has highlighted the key role of travel medicine consultations in Switzerland’s national measles elimination strategy (2011–2015). Analyzing data from the University of Zurich Travel Clinic between 2010 and 2016, researchers found that 11.6% of consultations resulted in measles vaccination, mainly prior to travel (90.9%). The majority of vaccinations (99.4%) involved adults as part of a catch‐up vaccination, and 13.6% of those eligible according to FOPH recommendations were vaccinated. These results highlight the importance of travel consultations in boosting measles vaccination coverage, as a complement to national vaccination programs [143].

The analysis revealed opposition to vaccination on part of the Romanian population, difficult to quantify, but strong enough to influence and hinder good healthcare practices. Romanian authorities should implement proactive strategies to promote the importance and safety of vaccination among the population, by conducting information campaigns in the media and taking measures to combat myths and misinformation. These efforts should also target marginalized or disadvantaged population groups. This study can help public health authorities and practitioners to better understand the population’s health information behavior and the profile of those influenced by antivaccination. It would also make it possible to identify and analyze the factors influencing people’s refusal to vaccinate [144].

Measles epidemics remain a major global health problem, particularly in low‐resource regions, despite the availability of safe and effective vaccines. The resurgence of measles underscores the crucial need to address the underlying health inequalities that hamper vaccine coverage, such as political instability, growing mistrust of all vaccines, economic collapse, and weak healthcare systems. Vulnerable populations, including unvaccinated children and pregnant women, are at increased risk of serious complications, particularly in areas where access to healthcare and vaccination services is limited. Persistent disparities in vaccine uptake, due to the social determinants of health such as ethnicity, socioeconomic status, and geographic location, exacerbate these risks. This underscores the need for equitable immunization strategies that ensure access to life‐saving vaccines for all, particularly in hard‐to‐reach areas [12].

Despite widespread vaccination, measles cases increased between 2010 and 2019, with a marked rise in 2019 (+140% compared to 2010). Analysis of WHO data shows that this resurgence is linked to several factors. In low‐ and middle‐income countries, variables such as gross national income, literacy, urbanization, and political stability influence measles incidence, reflecting access to public health infrastructure and vaccines. In contrast, in high‐ and upper‐middle‐income countries, vaccine hesitancy is a major factor in the resurgence of cases. These results underline the importance of adapting measles control strategies according to socioeconomic and regional contexts, in order to improve vaccination coverage and reduce transmission [145].

The measles/rubella surveillance system in Bantul has several structural weaknesses. No public health center uses it for decision‐making, and involvement of private facilities and the community is limited. Lack of networks and partnerships, as well as gaps in data collection on accessibility and flexibility, compromise its effectiveness. To improve this system, the health office should strengthen the involvement of private players, ensure better monitoring and evaluation, and aim for vaccination coverage of at least 95%, particularly in vulnerable areas. The WHO aims to eliminate measles and rubella in Southeast Asia by 2023, but in Indonesia, vaccination coverage has fallen by 6% and reporting of vaccine‐preventable diseases has dropped by 30% due to the pandemic. In Bantul, the measles–rubella positive rate increased by 16% in 2022, and a measles outbreak with 71 cases was reported in March 2023. To identify program shortcomings, a descriptive cross‐sectional study was conducted following the 2006 surveillance system evaluation guidelines. The assessment focused on the structure and attributes of the system in 18 public health facilities in Bantul. Fifty two health workers were interviewed using a semistructured questionnaire, and the results were classified into three categories: good, fair, and poor [146].

To combat pandemics and protect humanity, a variety of measures are essential. These include public health measures, infection control, medicines, global surveillance, and vaccines. The increasing digitization of health systems generates large epidemiological datasets that, when appropriately governed, may enhance measles surveillance and response planning. Carefully validated machine learning models can assist in identifying high‐risk districts, monitoring immunity gaps, and optimizing allocation of limited resources. Nevertheless, their effectiveness depends on reliable reporting systems, transparent governance frameworks, and sustained technical capacity, particularly in resource‐constrained settings [147].

Jayan and Alathur explore the growing influence of misinformation in the Government 3.0 era, focusing on its impact on vaccination campaigns. They highlight how misinformation on social networking sites could lead to vaccine hesitancy, using the measles–rubella vaccination campaign in India as a case study. They also highlight the sociopolitical, religious, psychological, and economic factors that contribute to this hesitancy. Finally, they highlight the crucial role of Government 3.0 in combating misinformation and improving the effectiveness of healthcare programs [148].

Preventive measures against measles include vaccination, case reporting, information, education and communication, and isolation of the patient after the onset of symptoms. Measles should be managed on an outpatient basis, except for cases with complications or other conditions requiring hospitalization, due to the high risk of intrahospital transmission. Nonserious cases of measles should be treated on an outpatient basis and isolated at home. At home, the patient must wear a surgical mask, contacts must be limited to immediate family members who have been vaccinated or have a history of measles disease, and contact must be avoided with pregnant women, unvaccinated infants or young children in the household, or anyone with an immune deficiency or on immunity‐reducing treatment. Children must stay away from school or must stay away adults from work for at least 5 days from the appearance of the rash, barring complications. Measles patients requiring hospitalization should be isolated from the onset of prodromal symptoms until 5 days after the appearance of the rash. Healthcare personnel in contact with these patients should use respiratory precautions during this period [33].

### 5.6. Political Recommendations

To eradicate measles, comprehensive health policies must be adopted that focus on promoting compulsory vaccination campaigns, particularly in areas where coverage rates are low. Governments must provide vaccines free of charge and ensure that they are easily accessible in health centers and schools [149]. Large‐scale awareness programs must be implemented to counter misinformation about the vaccine and boost community confidence in its efficacy [150]. In addition, epidemiological surveillance systems need to be strengthened to monitor cases and respond rapidly to epidemics [151]. International cooperation and the exchange of data and expertise between countries will play an essential role in achieving the goal of global measles elimination [152].

### 5.7. Actions Implemented by Morocco

The measures implemented in Morocco focused on strengthening national and regional public health coordination and response systems. They also involved the implementation of a national surveillance and response plan aimed at improving patient care, strengthening epidemiological monitoring, and supporting vaccination activities. A consolidated surveillance system was deployed to complement existing mechanisms. Coordination meetings and field support missions were organized to assist local teams. Laboratory capacities, as well as activities to verify vaccination status and conduct catch‐up campaigns, were also strengthened in primary healthcare facilities. A national campaign conducted from October 28, 2024, to November 17, 2024, in coordination with relevant sectors, supported the verification of children′s vaccination status and catch‐up efforts. Finally, communication initiatives were carried out to raise public awareness and promote vaccination [32]. In Morocco, local measures include the implementation of a point‐in‐time monitoring system in all schools in the region; the reporting of all unexcused absences to public health authorities, requiring every child to present their immunization record at the start of each new school year, particularly for elementary school students, as well as referring unvaccinated children or those with incomplete vaccinations to public health facilities to update their immunizations. They also include the implementation of a system for monitoring and reporting measles cases in all closed communities, with a referral pathway to care; a monitoring and surveillance system in facilities housing at‐risk populations (boarding schools, prisons, etc.); and a priority pathway for the management of measles cases. Organize an immediate response to notified cases of measles, and monitor the measles epidemiological situation and response actions weekly with the regional health and social protection departments [29].

## 6. Conclusion

Measles is an infectious viral disease that has been a persistent public health problem for decades. Despite global efforts, the complete elimination of measles remains elusive due to logistical obstacles, community resistance, and changing epidemiological factors. A review of historical and geographical approaches shows that mass vaccination campaigns have been the most effective in reducing infection rates, particularly in areas with high health coverage. However, problems remain, such as the uneven distribution of vaccines, the easy spread of the virus in densely populated areas, and the negative effects of measles‐induced immunosuppression. Advances in our understanding of immune mechanisms have demonstrated the essential role of innate and adaptive immunity in the fight against the virus. Strengthening herd immunity through effective vaccination is an essential tool for controlling epidemics. In addition, vaccine innovations such as inhalable vaccines and messenger ribonucleic acid–based vaccines offer promising solutions for improving the effectiveness and sustainability of immunization campaigns. Digital epidemiological tools, including machine learning–assisted risk modeling, may complement conventional surveillance systems when supported by robust data governance and infrastructure. Strengthening international cooperation through partnerships between governments, healthcare organizations, and the private sector can accelerate vaccination campaigns and improve healthcare infrastructures. In this context, political decision‐makers, researchers, and health authorities need to take more ambitious steps to accelerate efforts to eliminate measles. These include strengthening sustainable immunization programs, raising community awareness of the importance of vaccines, and developing evidence‐based strategies to address epidemiological challenges. Achieving a measles‐free world is not just a health goal; it is a moral obligation that requires concerted scientific, political, and societal efforts to ensure a healthier, more sustainable future for generations to come.

## Author Contributions

Abdelilah Merabti: conceptualization, methodology, formal analysis, investigation, writing–original draft, and writing–review and editing.

Youssef Miyah: conceptualization, methodology, formal analysis, investigation, writing–original draft, and writing–review and editing.

Mohammed Benjelloun: conceptualization, methodology, formal analysis, investigation, writing–original draft, and writing–review and editing.

Chadia Zahouani: writing–original draft, and writing–review and editing.

Wafaa Al Hassani: writing–original draft, and writing–review and editing.

Rajae Lamsyah: writing–original draft, and writing–review and editing.

Samia Essadki: writing–original draft, and writing–review and editing.

Laila Ihrai: writing–original draft, and writing–review and editing.

Saadia El Filali: writing–original draft, and writing–review and editing.

Anis Sfendla: writing–original draft, and writing–review and editing.

Mustapha Yaagoubi: writing–original draft, and writing–review and editing.

Chakib Nejjari: writing–review and editing and supervision.

## Funding

No funding was received for this manuscript.

## Consent

The authors have nothing to report.

## Conflicts of Interest

The authors declare no conflicts of interest.

## Data Availability

Data sharing is not applicable to this article as no datasets were generated or analysed during the current study.
